# Intelligent Aging Home Control Method and System for Internet of Things Emotion Recognition

**DOI:** 10.3389/fpsyg.2022.882699

**Published:** 2022-05-09

**Authors:** Xu Wu, Qian Zhang

**Affiliations:** ^1^School of Art and Design, Tianjin University of Technology, Tianjin, China; ^2^School of Control and Mechanical Engineering, Tianjin Chengjian University, Tianjin, China

**Keywords:** internet of things, emotion recognition, smart home, aging, MFCC

## Abstract

To solve a series of pension problems caused by aging, based on the emotional recognition of the Internet of Things, the control method and system research of smart homes are proposed. This article makes a detailed analysis and research on the necessity, feasibility, and how to realize speech emotion recognition technology in smart families, introduces the definition and classification of emotion, and puts forward five main emotions to be recognized in speech emotion recognition based on smart family environment. Then, based on this, it analyses the acquisition methods of emotional speech data. On this premise, this article discusses and analyses the related problems of voice data acquisition in smart homes, such as the voice characteristics and acquisition methods, puts forward three rules for voice text design, and determines the relatively suitable hybrid recording acquisition method applied in a smart home environment. At the same time, the design and establishment process of intelligent family emotional speech database is described in detail. The related problems of feature extraction in speech emotion recognition are studied. Starting from the definition of feature extraction, this article expounds on the necessity of feature extraction in the process of recognition and analyses the characteristics of the speech signals. For the specific environment of the smart family, the speech signal required to be processed needs to be close to the auditory characteristics of the human ears, and the speech signal contains enough information. Finally, the Mel frequency cepstrum coefficient (MFCC) is selected as the feature parameter applied in this article, and the extraction process of MFCC is introduced in detail.

## Introduction

Today’s society is facing two major trends: the gradual and rapid aging of population structure and the rapid development of science and technology. On the one hand, with the advent of the “white hair era,” the physical and mental health of the elderly is gradually weakened. With the process of modernization, many children are too busy to take care of the daily life of the elderly because of their busy study and work, resulting in the continuous increase in the number of empty nesters. However, due to certain limitations, social pension institutions cannot take into account the elderly in an all-round way, so many elderly people can only take care of the elderly at home. Such a way of life will inevitably bring a series of problems, which will bring great pressure to the social pension security and medical institutions. With the increase of the age of the elderly, the self-care ability of life is gradually weakened, and all kinds of accidents are easy to occur. How to ensure the quality of life and physical health of the elderly has become an important problem for the family and society (Sisavath and Yu, 2021). On the other hand, with the continuous development of microelectronics and intelligent technology, Internet of Things technology is regarded as the third-largest scientific and technological revolution wave in the world information industry after computer and Internet. With the continuous application of Internet of Things technology in the smart home system and its successful cases in the home field, it is necessary to apply the Internet of Things technology to the daily life and various life services of the empty nest elderly, which can solve various problems faced by society and the families to a certain extent. The radiofrequency technology adopted in the Internet of Things technology, that is, the electronic tag, embeds the electronic tag into the articles in the daily life of the elderly and the daily clothes of the elderly, carries out certain health status feedback on a daily behavior of the elderly, and implements the real-time monitoring function, so that children can quickly and accurately obtain the information fed back by the Internet of Things platform, master the health status of the elderly and put forward clear nursing suggestions (Nagayama et al., 2019).

**FIGURE 1:**
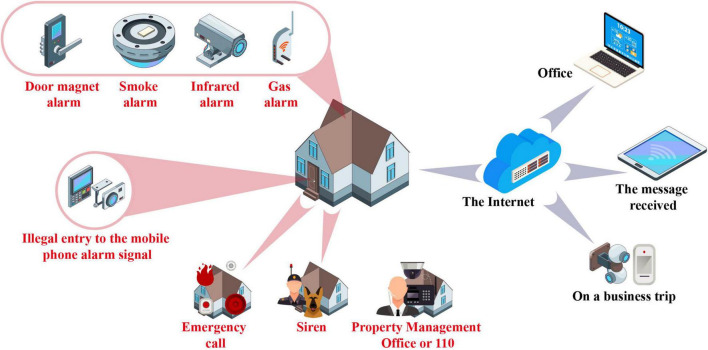
Intelligent aging home control.

Figure 1 shows the construction of intelligent aging home control.

## Literature Review

Zhang and Wang (2021) believe that with the rapid development of society, the pressure borne by the main family labor force is increasing. The implementation of the one-child policy has led to the obvious characteristics of family miniaturization, and it is difficult for children to bear the pension responsibility of the elderly. On the other hand, the ideas of the elderly also began to change, which prompted the transformation of the China’s pension model (Zhang and Wang, 2021). Meng (2020) believe that a simple social pension cannot fully meet the physical and psychological needs of the elderly. What we need to explore is a new pension model combining the advantages of home-based pension and social pension—a pension model dominated by home-based pension and supplemented by social pension (Meng, 2020). Su et al. (2019) said that in the recent years, many experts and scholars have put forward various pension models, including housing pension, hour care for the elderly, community pension, and so on. However, no matter what kind of pension mode we choose, as designers and the generation that will need to support the elderly, we need to focus on how to not only pay attention to the elderly’s own health, but also give them spiritual comfort, so as to create a pension life that can be satisfied both physically and psychologically (Su et al., 2019). Ahn et al. (2019) said that with the development of intelligent technology in society, the elderly have an increasingly strong desire to share their lives and improve their living standards, which is the most basic vision of life. In order to meet the expectations of the elderly, we must rely on the modern advanced intelligent technology to improve the quality of elderly care services (Ahn et al., 2019). Zhang et al. (2020) said that enabling elderly care is to apply advanced science and technology to the elderly care service platform to change the development direction of the elderly care model and improve the service level of home-based elderly care, community elderly care, and institutional elderly care. Jassim and Al-Hemiray (2019) believe that with the birth of emerging technologies such as digitization, networking, and intelligence, electronic devices have gradually moved toward the era of the Internet and intelligence. The development of intelligent pensions has become a new pension model with the trend of aging (Jassim and Al-Hemiray, 2019). Witzel et al. (2008) believe that the birth of the Internet of Things technology has turned people’s attention to “intelligent home-based elderly care.” Intelligent home-based elderly care is a new concept of home-based elderly care, which was first proposed by the British life trust, mainly through the implantation of chips in the living devices to make the home life of the elderly in a regulatory state. The purpose is to hope that the elderly can enjoy a high-quality elderly care life at home without being restricted by any factors (Witzel et al., 2008). Neme and Boiková (2013) believe that intelligent home-based elderly care relies on the Internet, big data, and cloud platform, and combines the physiological and psychological needs of the elderly to implant health technologies such as cloud medicine and big data into the elderly care system, improve the portability of medical equipment, establish electronic medical archives, real-time monitoring of body data, regular medication reminders, positioning to prevent the loss of the elderly, etc., help children to remotely pay attention to the living conditions of the elderly, get the physical condition data of the elderly at any time, and maximize the distance between the two sides (Neme and Boiková, 2013). Kim et al. (2020) believe that intelligent home-based elderly care not only improves the quality of life of the elderly in their later years, solves the problem of weakening elderly care resources, but also meets the psychological debt of children who cannot fulfill their elderly care obligations, which is in line with China’s “filial piety” culture. Beauchet et al. (2020) believe that the intelligent elderly care system integrates the experience and advantages of a variety of elderly care modes. It can be applied to diversified elderly care modes such as family elderly care, community elderly care, and institutional elderly care. It can provide rich elderly care services according to users with different life backgrounds and has high adaptability and adaptability. Therefore, intelligent technology is the basic and direction of the development of pension model in the future (Beauchet et al., 2020).

## Materials and Methods

**FIGURE 2:**
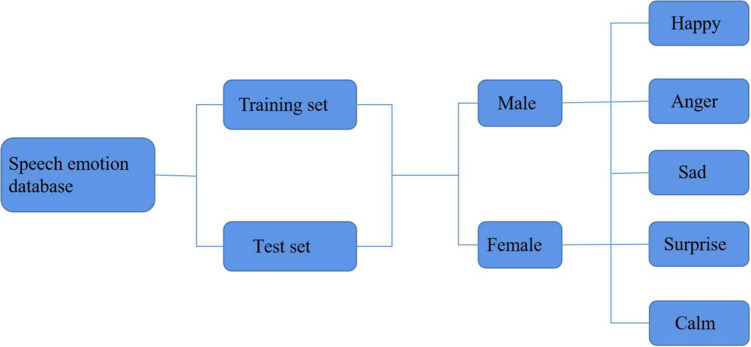
Voice emotion database structure.

Figure 2 shows the construction of the language emotion database.

### Language Emotion Feature Extraction

For the obtained speech emotion data, emotion recognition cannot be carried out directly. It needs to be transformed into processable data through feature extraction and then recognized through a specific-matching algorithm. This section introduces the speech emotion feature extraction in the smart family. At first, it makes a specific analysis on the relevant features of the speech emotion signal, and then on this basis, it extracts the speech emotion data features in the smart family. Here, MFCC parameters are selected as the feature vector and finally make a relevant summary.

#### Speech Emotion Signal Feature Analysis

**FIGURE 3:**
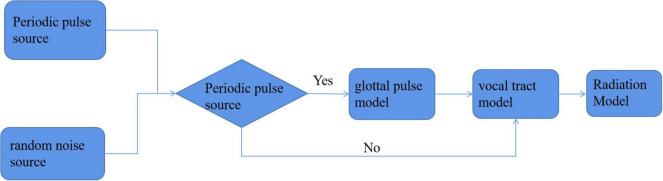
Excitation channel model.

Through the speech emotion database, we can get the speech files corresponding to each emotion, but we cannot recognize the emotion directly from these speech files. We must extract the corresponding speech features and match the emotional similarity from the features, so as to achieve the purpose of emotion recognition. Because speech emotion recognition should be based on the specific environment of the smart family, the feature parameters with low complexity, high representativeness, high stability, and good recognition rate should be considered in the feature selection of speech signals. Through the exploration and research of a large number of scholars, it is found that the generation of speech signals can be described by an excitation channel model. As shown in Figure 3, this model includes three submodels: excitation model, channel model, and radiation model. The excitation model mainly considers the signal source, which often has a certain random component. The phonetic features come from people’s vocal tract to a great extent. The radiation model simulates the speech features of lip expansion and divergence. It can be seen that the characteristic information contained in the speech signal is basically generated through the channel model part. In fact, in the daily life, people’s voice generally contains two kinds of characteristic information. One is linguistic information, that is, semantic information. For different languages, there are certain language norms. The information expressing the speaker’s purpose according to these norms is known as semantic information. The other kind of information is the acoustic information based on the channel model, which is also known as super semantic information. This kind of information cannot express the meaning of the language, but can convey other information of the speaker, such as current attitude, emotion, and accent belonging to a specific place. In general, acoustic information can be expressed from the following aspects (Pars et al., 2019):

(1) It refers to the acoustic characteristics related to the physiological structure used by people for phonation (such as energy, spectrum, resonance peak, and pitch peak), voice, nasal sound, respiratory sound, etc.
(2) It is the intonation, volume, rhythm, speed, and other characteristics influenced by elders in the process of learning to speak.
(3) It is the law of rising and falling tones and the characteristics of specific vocabulary pronunciation brought by people’s education level, the influence of surrounding non-relatives, and the overall local pronunciation environment.

Only from the perspective of the information transmitted by speech, it can be found that emotion can be expressed through the semantics of speech, as well as through the emotional attitudes contained in acoustic information. However, the emotion expressed through semantics is often very deceptive, which originates from the uncertainty and randomness of semantics itself. For example, the sentence “I like you so much” is simply to express my admiration for each other in terms of semantics; but in fact, it is possible that the speaker is in a mood of resentment and dissatisfaction, which actually means the opposite. It can be seen that the emotional state cannot be determined without the semantics of the specific environment. Therefore, the recognition of emotion in this article will only be analyzed from the acoustic characteristics. Usually, when people express their feelings through speech signals, they often change in speech rhythms such as speech rate, tone, and volume. For example, when people are in a sad mood, they speak slower, their tone decreases, and their voice is almost hard to hear. Through these relatively intuitive changes, we can find the causes of these changes. Table 1 summarizes the mapping relationship between some emotion and speech feature parameters. Generally, when people show a happy emotional state, the amplitude and intensity of speaking will focus on the last two words of a sentence, and the tone of the whole process will be higher than usual. In terms of speaking speed, there will be a significant acceleration. The speed of speaking is usually not known clearly, and there will be differential speed performance in the different situations. Often, there is a breathing sound in the sound. For the pronunciation of a sentence, the speed of the middle and first parts will speed up. Under the joint constraints of grammatical rules and physiological reasons, the words that are not particularly key in the sentence will become the middle transition of two adjacent intonations, and the tone of the exclamation at the end of the sentence will rise first and then decrease. From the perspective of speech clarity, people in this state will pay great attention to other people’s auditory experience, so they will be clearer when they are relatively calm. When the people show anger, it can be found through daily experience that people will be accompanied by physiological conditions such as faster heartbeat and higher blood pressure. This directly affects the changes of some speech parameters. First, the amplitude intensity will be significantly higher, and the speaking speed will be faster than that in the normal state. Similar to the happy state, there will be a large number of breathing sounds, and even chest sounds will increase the proportion in speech. When speaking, the emphasis on adverbs and verbs is obviously strengthened. Not only that, but the usual soft exclamation will also change to the upper tone.

**TABLE 1:** Mapping of speech feature parameters and emotion.

Type	Happy	Get angry	Sad	Calm	Pleasantly surprised
Strength	High	High	Low	Normal	High
Definition	Clear	Vague	Vague	Normal	Normal
Speed of speech	Fast or slow	Soon	Slow	Normal	Slightly faster
Average pitch	High	Very high	Low	Normal	Slightly higher
Pitch breadth	Wide	Wide	Narrow	Slightly narrow	Slightly wider
Sound quality	Breathing, harsh tones	Chest sound and breathing sound	Resonant sound	–	Breathing sound

Different from happy and angry, sad emotion belongs to an inhibitory emotional state. People in this state often show the characteristics of low tone and slow speech. This is because the pronunciation interval of two adjacent words is relatively large. At the same time, the nasal sound will be mixed in the speech, which indirectly leads to the vagueness of speech. As an internal perception, emotion has a very important feature that depends on regional factors such as family environment, social environment, humanistic environment, and geographical environment. Each country and nation has its own culture and living habits. This long-term influence leads to differences in the way of expressing emotion. Because the speech emotion database built in this article takes Chinese as the basic language, and the unique tone of Chinese determines the suprasegmental characteristics that play a great role in speech processing and emotion information analysis, such as time sequence structure, rhythm, pitch, and intensity. Therefore, we will mainly analyze some characteristics of Chinese (Guyon, 2019).

##### Fundamental Frequency Analysis

Fundamental frequency, i.e., fundamental audio rate. The whole process of opening and closing vocal cords is known as a pitch period and its reciprocal is the pitch frequency. It determines the pitch of tone and is one of the most common and important parameters in speech signal processing. The thickness, size, and relaxation of vocal cords determine the degree of the fundamental frequency. In general, the thinner, larger, and tighter the vocal cord, the higher its pitch frequency. Pitch frequency contains a lot of information in an emotional speech. Usually, the pitch frequency is calculated according to the definition, that is, the pitch period is calculated first, and then the reciprocal is the pitch frequency. Because the pitch period is uncertain, it can only be obtained by estimating the short-term average. So far, there is no unified standard for estimating pitch period in general. Different speakers need to be tested separately according to the statistical methods for different environments. Because of the difference of physiological structure, the frequency-based of men and women also show different ranges. Among them, 90% of men have a fundamental frequency range of 112–217 Hz, while a considerable number of women have a fundamental frequency range of 167–331 Hz. The pitch frequency will also change according to the change of emotional state, which is also the reason why it can be used as the characteristic parameter of emotion recognition.

##### Amplitude Analysis

The amplitude of speech can also be known as amplitude energy, which will show corresponding strength with the change of emotional state. In fact, the external expression of this feature is very common. It will be found in daily life that when people’s mood changes to an angry state, their voice will suddenly become high-pitched; when the people’s mood is sad, they often cannot hear the sound clearly. Actually, this is the function of amplitude energy. In short, amplitude energy represents the loudness of the sound. Generally, people’s perception of sound loudness depends on their sensitivity to speech signals of various frequencies. In addition to the general amplitude energy of speech signal, this feature will change accordingly when people are in a certain emotional state. When in the state of anger, surprise, and happiness, the corresponding speech amplitude energy is relatively high. On the contrary, for the emotional state of sadness and calm, people often show speech characteristics of low-amplitude energy. In terms of gender, the amplitude energy of women is still slightly higher than that of men, but the overall trend is not very different. At the same time, in terms of change rate, emotional states with an obvious tendency (such as anger and surprise) change faster.

##### Time Analysis

**FIGURE 4:**
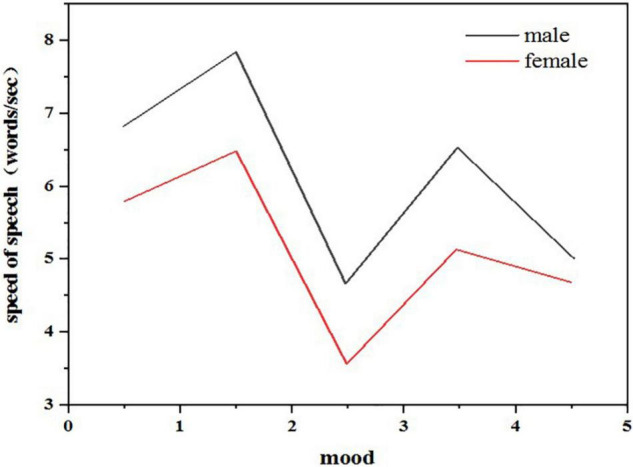
Comparison of speech speed of each emotion.

**FIGURE 5:**
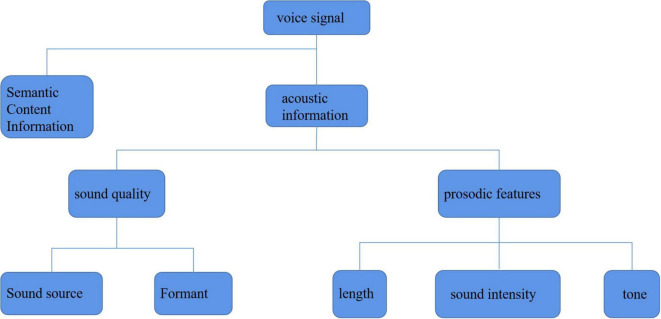
Classification of affective speech features.

The analysis of the time characteristics of the speech signal is mainly to analyze the characteristics of speech speed. As described in the previous chapter, the speech speed (speaking speed) will be very different under different emotional states. When people are in an angry emotional state, their speed of speaking is often the fastest, while when they are happy and surprised, their speed of speaking will also increase slightly. In addition, when they are calm and sad, their speed of speaking is relatively slow, which seems to be consistent with the real situation in daily life. From the perspective of men and women, women’s speaking speed is slower on average, but generally speaking, the characteristics of speaking speed are relatively consistent. As shown in Figure 4, after studying the feature analysis of speech signals, it can be found that there are many ways to recognize emotional information through speech. Generally, the acoustic feature information of speech signal is divided into two categories: sound quality feature and prosodic feature. Figure 5 shows the classification of characteristic information of speech signals. Sound quality characteristics: sound quality characteristic parameters reflect the shape change of glottic waves in the process of the speaker’s pronunciation. Among them, the pressure in the center of the vocal tract, the muscle expansion related to vocalization, and the vocal tract strength tension will affect the results of sound quality characteristic parameters (Batalla and Gonciarz, 2019).

###### Prosodic Feature

Prosodic feature refers to the change of sound length and intensity in speech signal except for the sound quality feature, which is also known as “suprasegmental feature” or “suprasound quality feature.” From the aspect of the sentence, the change of pitch, intensity, and pitch will form intonation; from the aspect of a syllable, the change of pitch will form “tone;” from the aspect of the phoneme, the change of length will form “long and short tones” with the function of discrimination; from the aspect of syllable combination, the change of factors such as sound intensity will form light stress. It can be seen that the prosodic characteristics of speech signals are consistent with the sound intensity and tone rise and fall of people’s speech at ordinary times. Therefore, the internal information of the speaker’s emotion will and attitude can be obtained directly through prosodic features. The prosodic feature of speech signal has also become the main feature information to be advanced in the research of speech emotion recognition.

#### Speech Emotion Feature Extraction

The ultimate goal of speech signal feature extraction is to obtain the feature data that can be processed by the specific model of computer application. For different application environments, there may be differences in the selection of features in the process of speech emotion recognition. For example, a feature may or may not be selected, even if it is deliberately strengthened or weakened. For example, for the “source” feature, its influence will be diluted or even ignored in the application of lie detection in the field of security. That is, for the emotional state to be recognized, the influence of different speakers is not considered. In a word, the system hopes that for any type of source, after the speech signal is collected from it, it can get good speech emotion recognition. Therefore, for the feature extraction of speech emotion recognition, it is meaningful to select appropriate features according to the different scenes. Feature extraction of the speech signal is the premise that real speech information can be recognized. More vividly, it sets up an interworking bridge between speech emotion data and the recognition module. In specific applications, it makes the feature parameters more comprehensive for the expression of information contained in speech signal as far as possible, which will be closer to the real situation. In addition, whether a good recognition rate can be achieved is also an important consideration standard. Under the condition of ensuring the authenticity and recognition rate, the feature extraction algorithm with simple calculation and good time complexity will be the selection standard.

In general, the feature extraction of speech emotion recognition mainly includes the following steps:

(1) Digital signal processing. This process mainly includes antialiasing filtering, sampling, A/D conversion, etc., and it is mainly to turn the analog signal of voice into a digital signal that can be processed by computer.
(2) Pretreatment. In general, there are pre-emphasis, windowing, and framing. This is to reduce the noise interference in the speech signal and strengthen the useful speech information at the same time.
(3) Parameter extraction. This is the core of feature extraction. In the processed speech signal, through this step, the parameters expressing the emotional characteristics of speech can be obtained (Akour, 2020).

## Results and Analysis

According to the specific application environment of the smart family, based on the research and comparison of a large number of literature, and considering the authenticity, amount of information, and time efficiency, this article finally selects the MFCC algorithm as the core algorithm of speech emotion feature extraction. According to the aforementioned content, the overall process of speech emotion recognition feature extraction based on the smart home environment can be summarized as the following main steps: digitization of speech signal, preprocessing process, extraction of specific feature parameters, etc. Each step contains many small steps, which will be described in detail later.

**FIGURE 6:**
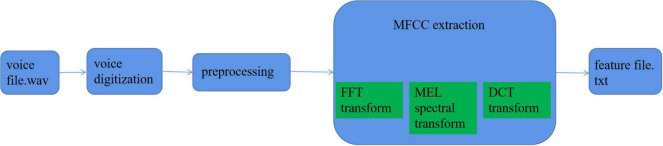
Feature parameter extraction process.

Figure 6 shows the specific extraction process.

### Speech Signal Digitization

**FIGURE 7:**
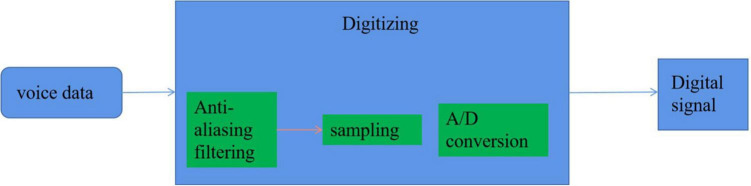
Digital voice signal.

Before the feature extraction of the speech signals, it must be digitally converted into a digital signal that can be recognized by the computer. For the smart home environment, the digitization of speech emotion recognition mainly includes three steps: antialiasing filtering, sampling, and A/D conversion, as shown in Figure 7.

#### Antialiasing Filtering

The general speech signal frequency is mainly between 80 and 3,400 Hz, but the recorded speech signal data will have 50 Hz power frequency and high-frequency interference above 7,000 Hz. Moreover, because of the interference of noise, the speech signal is in a mixed superposition interference. In order to reduce the distortion caused by aliasing and the interference of a large amount of noise, the speech signal should be filtered. To achieve this goal, the selected filter must be a band-pass filter. For most encoders, the upper bound frequency is 3,400 Hz and the lower bound frequency is 60 Hz (Prasetyo et al., 2019).

#### Sampling

Generally speaking, it is to convert a continuous signal into a discrete signal. Here, it is to convert the speech analog signal with continuous amplitude value and time value into a discrete analog signal with discrete time and continuous amplitude value. According to Shannon’s theorem, in order to avoid information loss as much as possible, the sampling frequency is generally 8,000 Hz.

#### Analog-to-Digital Conversion

The sampled data have been discretized in time. In this step, the amplitude value will be quantified and represented by a bit sequence, so as to obtain the speech digital signal. For any analog-to-digital conversion process, only a limited number of binary codes can be used to quantify. For speech signals, the word length of 8, 12, 16, and 20 bit is generally selected. There are two models: non-linear converter and linear converter. The non-linear converter is generally 8 bits, which corresponds to the 12-bit linear converter often used at present. Sometimes, in order to facilitate processing, it is usually necessary to convert 8-bit non-linearity into 12-bit linearity.

### Pretreatment

**FIGURE 8:**
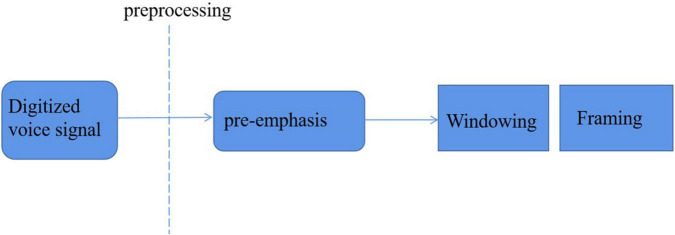
Speech signal preprocessing.

After digital processing, voice signals are sequentially stored in a data area in the form of a sequence. Generally, the way of the circular queue is adopted, which can ensure that a large number of voice data can be met at the cost of limited capacity storage. Because the speech feature parameter is a non-stationary state, which changes continuously with time, but it is a steady-state process in a short time. Therefore, a preprocessing process is needed between the extraction of feature parameters, as shown in Figure 8.

#### Pre-emphasis

When the frequency increases, the power of speech signal will generally decrease, and the signal-to-noise ratio of most high-frequency ends will decrease sharply, while the energy is concentrated in the low-frequency part. To make the high frequency and low frequency have the same signal-to-noise ratio and maintain the stability of the whole frequency band, pre-emphasis processing is required. Its purpose is to improve the high-frequency part and make the spectrum of speech signal flat, so as to calculate the spectrum with the same signal-to-noise ratio in the whole frequency band, which is conducive to the analysis of channel characteristics (Hasan et al., 2019).

Pre-emphasis generally makes the speech signal pass through a first-order high pass digital filter. The formula is:


(1) $$H\left(Z\right)=1-a\times Z^{-1}$$

Where a is the weighting coefficient, which is infinitely close to 1, generally between 0.9 and 1.0.

#### Framing

In order to avoid the complexity of the overall processing of speech signal, it is divided into frame segments for processing according to the short-term nature of speech. Frame division generally adopts the method of intermittent overlapping frame division, that is, the frame without overlapping area is obtained first, but this often makes the frame too independent. In order to maintain a smooth transition, there is an overlapping area between the front and rear frames, which is usually called frameshift and its size generally does not exceed half of the whole frame length.

#### Windowing

In the process of framing, the voice signal is windowed. A window filter is added to the speech signal. The process of windowing is to intercept the speech signal by using the truncation function. Different window functions and appropriate window length may be required for different speech feature parameters extraction. The two commonly used window functions are rectangular window and Hamming window.

The calculation formula of the rectangular window is (Lee et al., 2021):


(2) $$W\left(n\right)=\left\{ \begin{array}{c} 1,0\leq n\leq \left(N-1\right)\\ 0,n\mathrm{is}\mathrm{other}\mathrm{value} \end{array}\right.$$

The calculation formula of Hamming window is:


(3) $$W\left(n\right)=\left\{ \begin{array}{c} 0.54-0.46\cos\left[\frac{2\pi n}{N-1}\right],0\leq n\leq \left(N-1\right)\\ 0,n\mathrm{is}\mathrm{other}\mathrm{value} \end{array}\right.$$

It can be seen from the formula that the rectangular window has relatively simple operation requirements and good smoothness for the spectrum, but the side lobe is relatively large and the high-frequency part is lost. For the short frame length, this disadvantage is more and more obvious. The main lobe width of Hamming window is large, which can reduce the leakage of the spectrum.

The selection of window length can be analyzed by the following formula:


(4) $$\Delta f=\frac{1}{N}T_{s}$$

Where Δ*f* is the resolution of frequency, *N* is the window length, and *T*_*s*_ is the sampling period.

**FIGURE 9:**
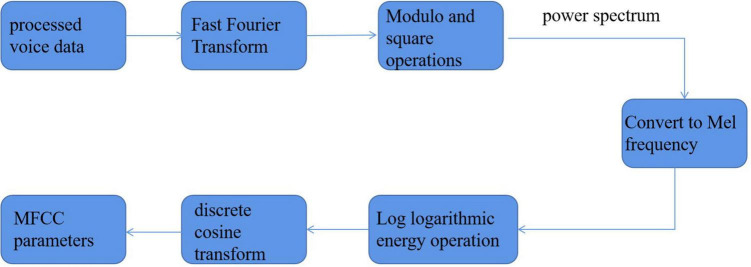
The extraction process of MFCC parameters.

It can be seen that when the sampling period is fixed, the smaller *n* is, the time resolution is improved, but the frequency resolution is reduced. It seems to be contradictory, so it is necessary to select the appropriate window length according to the actual situation. For the smart family environment, this article will choose a more appropriate Hamming window in the window shape. In the selection of window length, because it needs to take into account different ages and genders from children to the elderly, from women to men, and the span range of frequency is relatively large, so 256 window lengths will be selected. After a series of digital processing and preprocessing, the specific feature extraction algorithm will be used for parameter extraction. Combined with the analysis of the previous content, the Mel frequency cepstrum coefficient (MFCC) will be extracted in this article. The specific process is shown in Figure 9.

To make the result of speech analysis closer to the feeling of the human ear, Mermelstein and Davies proposed a new cepstrum parameter, namely, the Mel frequency cepstrum coefficient. It belongs to the category of cepstrum analysis of speech signals. Cepstrum analysis originated from a new concept proposed by Healy et al. (1963): the “cepstrum” of the logarithmic spectrum. The purpose of cepstrum analysis of speech signals is to obtain cepstrum characteristic parameters for speech recognition and processing (Pimenta and Chaves, 2021).

However, different from the general actual frequency cepstrum, MFCC analyses the auditory characteristics of the human ear and expresses a common corresponding relationship from the speech signal frequency to the actual “perceived” frequency of the human ear because the size of the sound really heard by the human ear is not in a complete positive proportion to the frequency of the actual sound. Therefore, the measurement of Mel frequency is more in line with the auditory characteristics of human ears. The relationship between Mel frequency and actual frequency can be expressed by the following equation:


(5) Mel(f)=2,595g(1+f/700)

**FIGURE 10:**
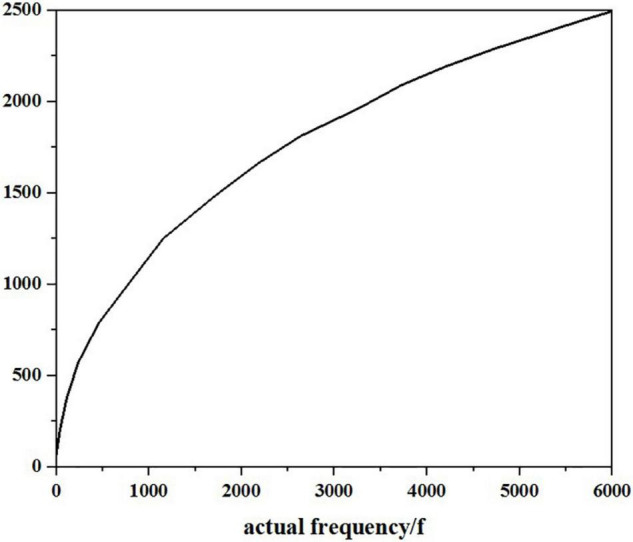
Relationship between actual frequency and Mel frequency.

Here, the unit of the actual frequency f of sound is Hz. After research, the growth of Mel frequency and actual frequency is roughly the same, and it is a linear growth state below 1,000 Hz; when it is higher than 1,000 Hz, it shows a logarithmic distribution. As shown in Figure 10, the initial understanding of speech signal is the process of transmission and penetration of acoustic energy in the medium, showing a continuous distribution state on the time axis. Therefore, the earliest research on speech signals began with the analysis of the time domain, but it is difficult to realize this way through continuous experiments. However, the field of the frequency domain, which is later than that of the time domain, has made great progress, and it has been proved that the frequency domain is better realized. Therefore, many methods such as Fourier transform are used to transform the time domain to frequency domain. Mel frequency cepstrum coefficient (MFCC) is obtained by changing the spectrum of the speech signal from general frequency scale to Mel frequency scale in its frequency domain and then transformed to cepstrum domain. When calculating the Mel frequency cepstrum coefficient, after the Fourier spectrum analysis of speech signal, the physical frequency scale Hz is converted into psychological frequency scale Mel through Mel scale filter bank. After taking the logarithm, the Mel frequency cepstrum coefficient is obtained through discrete cosine transform. In the process of MFCC extraction, we must first get several intermediate values: short-term average energy, short-term average amplitude, and short-term zero-crossing rate. Because the speech signal is not in a stable state, the method of processing steady-state signal is not suitable for processing speech signal. However, through the observation of speech signals, it is found that it presents a relatively stable process in a short time of 10–30 ms. Therefore, it is short term.

Short time average energy refers to the average energy value of the speech signal itself in a short time interval. The calculation formula is:


(6) $$E_{n}=\sum_{m=0}^{N-1}x_{n}^{2}\left(m\right)$$

The short-time average amplitude is a supplement to the short-time average energy. Because the short-time energy is calculated based on the square, it is very sensitive to the high-level state level. The application of short-time average amplitude will not cause a large difference due to the calculation of the quadratic power of the sampling value. The calculation formula is:


(7) $$M_{n}=\sum_{m=0}^{N-1}\left|x_{n}\left(m\right)\right|$$

The function of short-time average energy and short-time average amplitude in speech signal processing: It can distinguish voiced area from the voiced area; it can separate vowels and initials, and divide the sound area and silent area.

Short time zero-crossing rate refers to the number of times that the waveform curve of a time frame intersects the abscissa axis in the speech signal. It is the simplest kind of analysis. Generally, the number of symbol transformations of adjacent sampled values of discrete signals is called zero crossing; the zero crossing of the continuous signal represents the time-domain waveform passing through the time axis. The function of short-time zero-crossing rate is to remove the influence of external noise and find useful speech signals. The spectrum of speech signals can be generally estimated.


(8) $$Z_{n}=\frac{1}{2}\sum_{m=0}^{N-1}\left|sgn\left[x_{n}\left(m\right)-sgn\left[x_{n}\left(m-1\right)\right]\right]\right|$$

#### Fast Fourier Transform

Developed by Cooley and Tuki based on discrete Fourier transform, it uses digital calculation for spectrum analysis. The Fourier transform of continuous time function should be rewritten into the transformed relationship of the discrete-time waveform before using the fast Fourier transform. The core of realizing Fourier transform is to divide the one-dimensional array into two-dimensional matrix processing by using the periodic characteristics of the decomposed speech signal.

For a finite time series {*X*(*n*)}, the Fourier transform of 0≤*n*≤*n*−1 is defined as:


(9) $$X\left(k\right)=\sum_{n=0}^{N-1}x\left(n\right)e^{-j\left(\frac{2\pi }{N}\right)nk},k=0,1\ldots N-1$$

Where $e^{-j\left(\frac{2\pi }{N}\right)nk}$ is the period.

The basic algorithm requirement of fast Fourier transform is to divide the original length *n* series into two relatively short series. That is, the odd sequence *x*_1_(*n*) = *x*(2*n* + 1)and the even sequence *x*_2_(*n*) = *x*(2*n*), which are transformed and added, respectively. It can be seen that there will be the following relationship:


(10) $$X\left(k\right)=X_{1}\left(k\right)+{\left[e^{-j\left(\frac{2\pi }{N}\right)}\right]}^{k}X_{2}\left(k\right)$$

#### Modular Operation and Square Operation

According to the calculation results of fast Fourier transform, carry out modular operation first, and then square it. Thus, a discrete power spectrum can be obtained.

#### Mel Frequency Conversion

**FIGURE 11:**
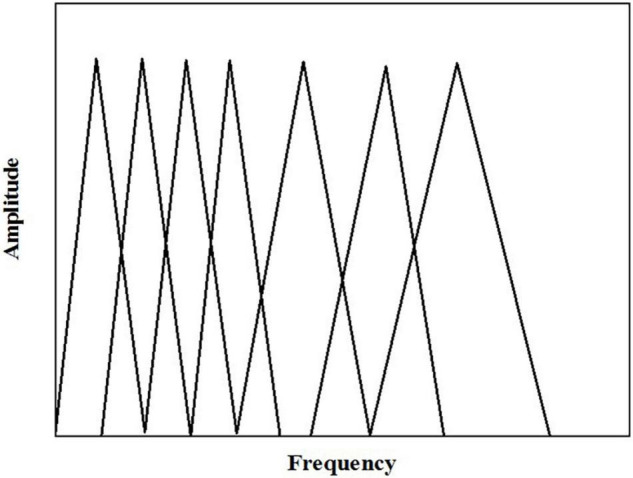
Mel triangular filtering.

This process is to convert normal frequency into Mel frequency measurement. Specifically, the discrete power spectrum obtained earlier is filtered by a set of triangular filter banks measured by Mel scale. Here, *m* (determined by the cut-off frequency of the speech signal) triangular filters with a center frequency of *f*(*l*) and an *l* value from 1 to *m* are defined, in which the adjacent filters in the position are overlapped. With the increase of filter serial number, the central frequency of each filter begins to increase, but it is equidistant on the Mel axis, as shown in Figure 11.

Mel frequency after triangular filtering meets the following requirements:


(11) $$H_{1}\left(k\right)=\left\{ \begin{array}{c} 0,k<f\left(l-1\right)\text{or}k\geq f\left(l+1\right)\\ \frac{2\left(k-f\left(l-1\right)\right)}{\left(f\left(l+1\right)-f\left(l-1\right)\right)\left(f\left(l\right)-f\left(l-1\right)\right)},f\left(l-1\right)\leq k\leq f\left(l\right)\\ \frac{2\left(f\left(l-1\right)-k\right)}{\left(f\left(l+1\right)-f\left(l-1\right)\right)\left(f\left(l+1\right)-f\left(l\right)\right)},f\left(l\right)\leq k\leq f\left(l+1\right) \end{array}\right.$$

Of which: $\sum_{l=0}^{L-1}H_{l}\left(k\right)=1.$

#### Log Logarithmic Operation

Logarithmic operation is performed on the obtained power after filtering to obtain:


(12) $$L_{1}=\ln\left(\sum_{k=0}^{N-1}{\left|X_{a}\left(k\right)\right|}^{2}H\left(k\right)\right),0\leq l\leq M$$

#### Discrete Cosine Transform

After the logarithmic result obtained in the previous step, perform discrete cosine transform (DCT), and then transform the result to cepstrum domain to obtain *C*_*mfcc*_(*k*), where k is from 1 to L-1.


(13) $$C_{mfcc}\left(k\right)=\sum_{l=0}^{M-1}L_{1}\cos\frac{\left(2l-1\right)k\pi }{2M}$$

Where *k* = 1, 2, L.

In this way, an L-dimensional coefficient is obtained. Generally, 12–16 is selected. Generally, when *k* = 0, it represents the DC part and needs to be rounded off.

In this experiment, the sampling frequency is 8,000 Hz, the number of filters is 25, the size of sound window is 256, and the frame shift is 128. As shown in Table 2, the time used to extract 12–16 dimensional feature data, respectively. It can be seen from Table 2 that in the smart home environment, in order to save the system processing time as much as possible and fully obtain the multidimensional characteristic parameters.

**TABLE 2:** Time taken for parameter extraction of different dimensions (MS).

Dimension	12	13	14	15	16
Time	142	149	162	184	195

## Conclusion

At first, this article takes the establishment of voice emotion database as the goal, successively discusses the definition and main classification of emotion in the field of smart family, and determines that happiness, sadness, anger, calm, and surprise are the main emotions objectives identified in this article. After analyzing the establishment of a relevant voice emotion database, a hybrid voice emotion database based on a smart home environment is proposed, and the database needed in this article is established. Then, the emotional speech features are deeply introduced and analyzed. Through comparison, the relatively appropriate MFCC feature parameters based on the smart home environment are determined. Then, the steps of feature extraction are introduced from general to detailed analysis. On this basis, the MFCC feature parameters are extracted through experiments. With the progress of information technology and the continuous popularization of scientific and technological products, great changes have taken place in people’s daily life. In addition to the pursuit of material life, everyone also requires emotional care. At first, the concept of the smart family only made people feel convenient in material terms. With the development of artificial intelligence and emotional computing, it has become an inevitable trend to make smart family have the function of emotional recognition and provide services from the perspective of “people.” The key to introducing emotion recognition into the smart home system is to make this technology suitable for the situational environment and have the characteristics of feasibility, effectiveness, and convenience. Considering the aforementioned aspects, emotion recognition through speech signals has become an inevitable choice. In addition to the popularity and easy capture of speech, a large number of studies in this field also provide a reference for speech emotion recognition based on a smart home environment.

## Data Availability Statement

The original contributions presented in the study are included in the article/supplementary material, further inquiries can be directed to the corresponding author.

## Author Contributions

All authors listed have made a substantial, direct, and intellectual contribution to the work, and approved it for publication.

## Conflict of Interest

The authors declare that the research was conducted in the absence of any commercial or financial relationships that could be construed as a potential conflict of interest.

## Publisher’s Note

All claims expressed in this article are solely those of the authors and do not necessarily represent those of their affiliated organizations, or those of the publisher, the editors and the reviewers. Any product that may be evaluated in this article, or claim that may be made by its manufacturer, is not guaranteed or endorsed by the publisher.
